# Association between Obesity and Cardiovascular Disease Risk Factors in Different Age Groups of Adolescents: An Analysis of Data from the Korean National Health and Nutritional Examination Survey

**DOI:** 10.3390/children10050827

**Published:** 2023-05-01

**Authors:** Joowon Lee, Seul Gi Cha, Jue Seong Lee, Susan Taejung Kim, Young Hwan Song

**Affiliations:** 1 Department of Pediatrics, Seoul National University Bundang Hospital, Seongnam 13620, Republic of Korea; 2 Department of Pediatrics, Asan Medical Center, University of Ulsan College of Medicine, Seoul 05505, Republic of Korea; 3 Department of Pediatrics, Korea University Anam Hospital, Seoul 08308, Republic of Korea; 4 Department of Pediatrics, Seoul National University Children’s Hospital, Seoul 03080, Republic of Korea

**Keywords:** heart disease risk factors, overweight, obesity, adolescent

## Abstract

We investigated the association between obesity and cardiovascular disease risk factors (CVDRFs) in adolescents. We performed a cross-sectional study using the data from 8149 adolescents, aged 10–18 years, included in the Korean National Health and Nutrition Examination Survey (2011–2020). Using the body mass index, we defined “overweight” (≥85th to <95th percentile) and “obese” (≥95th percentile). We analyzed the associations between obesity and CVDRFs (high blood pressure, abnormal lipid profiles, and high fasting glucose levels) by sex and age groups (early [10–12 years], middle [13–15 years], and late [16–18 years] adolescence). When analyzing all the subjects, being overweight was correlated with high blood pressure and abnormal all-lipid profiles in boys and high triglyceride and low high-density lipoprotein cholesterol levels in girls, while obesity was associated with all CVDRFs in both boys and girls. Analyzing separately in the age subgroups, the correlation between obesity and CVDRFs tended to be shown earlier in boys than in girls, and obesity tended to be associated with CVDRFs earlier than being overweight. The association between obesity and CVDRFs may begin to be shown at different periods of youth, depending on the degree of obesity, CVDRF variables, and sex.

## 1. Introduction

Recently, obesity in children and adolescents has become a significant global health issue [1]. The prevalence of obesity among children and adolescents has increased over the past several decades globally [2,3,4]. On the basis of developing evidence, it is apparent that adiposity in children and adolescents increases the likelihood of becoming obese as adults and is connected to a higher risk of cardiovascular morbidity and mortality, including stroke, congestive heart failure, ischemic heart disease, and cardiovascular death in adulthood [5,6,7,8,9]. Additionally, many studies have reported that children and adolescents with obesity had a higher prevalence of cardiovascular disease risk factors (CVDRFs) such as high blood pressure, abnormal lipid profiles, and high fasting glucose than normal weight children and adolescents of the same age group [10,11,12,13,14,15]. 

However, little is known about the precise age at which the relationship between obesity and CVDRFs begins. In order to identify the onset age of this relationship, longitudinal studies on young children are required but have been scarcely performed. Instead of longitudinal studies, there have been a few studies which could infer the age pattern of the relationship between obesity and CVDRFs by analyzing the nationwide-scale cross-sectional data even though they have some shortcomings. A population-based study by Lambert et al. revealed an association between weight status and CVDRFs by age groups without considering sex difference [16]. In a study using the National Health and Nutrition Examination Survey conducted by Skinner et al., the relationship between the degree of obesity and CVDRFs by age groups was investigated without comparison with a normal weight control group [17].

In the present study, we investigated the association between obesity and CVDRFs in Korean adolescents using the nationally representative data in order to identify the period at which this association begin to be shown with consideration of CVDRF variables, degree of obesity, and sex.

## 2. Materials and Methods

### 2.1. Study Population

The Korean National Health and Nutrition Examination Survey (KNHANES) is a nationwide cross-sectional survey with a stratified and multi-stage clustered sampling design that comprises a health interview, a nutritional survey, and a health examination including anthropometric and laboratory data. The Korean Centers for Disease Control and Prevention (KCDC) approved the KNHANES and collected data after obtaining written informed consent from all participants. A detailed description of KNHANES has been published elsewhere [18].

This study included KNHANES participants from 2011 to 2020 who were 10–18 years of age at the time of the survey and examination. The participants who had congenital heart disease, renal failure, or diabetes mellitus were excluded. A total of 8149 adolescents (4314 boys and 3835 girls) were included in the present study. We classified the participants into three age groups: early adolescence (10–12 years of age), middle adolescence (13–15 years of age), and late adolescence (16–18 years of age).

### 2.2. Assessments of Dietary Intake and Phisical Activity

In an in-person interview, dietary intake was measured using a food intake questionnaire with a 24-hour dietary recall approach. The formula used to calculate the energy and macronutrient intakes from the food the participant had consumed the day before was as follows: total energy/nutrient intake = intake frequency x food intake amount x energy/nutritent by food item. Daily intake of energy, protein, fat, carbohydrate, and sodium were computed. The physical activity was evaluated using the International Physical Activity Questionnaire (IPAQ) [19]. We estimated weekly physical acitivity time for walking, moderate exercise, and vigorous exercise (hour/week).

### 2.3. Anthropometric and Laboratory Measurements

Anthropometric measurements were performed by trained examiners. Height was measured using a stadiometer (Seca 225 in 2011 to June 2019, Seca 274 in July 2019 to 2020; Seca, Hamburg, Germany) in 0.1 cm increments. Weight was measured using an electronic balance (GL-6000-20; G-tech, Seoul, Korea) in 0.1 kg increments. Body mass index (BMI) was computed as weight (kg) divided by height squared (m^2^). The BMI z-scores adjusted for age and sex were calculated based on the KCDC reference data [20]. Blood pressure was measured on the right arm with the participants in a sitting position and at rest for at least 5 min, using a mercury sphygmomanometer in 2011–2019 (Baumanometer Desk Model 0320 in 2011–2012, Baumanometer Wall unit 33(0850) in 2013–2019; W.A. Baum, Copiague, NY, USA) or a mercury-free electronic sphygmomanometer in 2020 (Greenlight 300; Accoson, Ayrshire, UK). Three measures were obtained from all participants using an appropriately sized cuff and the average values of the second and third systolic blood pressure (SBP) and diastolic blood pressure (DBP) were reported.

Blood samples were obtained in the morning after an overnight fast and analyzed in a national central laboratory. The serum levels of total cholesterol, triglyceride (TG), high-density lipoprotein cholesterol (HDL-C), and glucose were measured using Hitachi Automatic Analyzer 7600 (Hitachi, Tokyo, Japan) in 2011–2012, Hitachi Automatic Analyzer 7600-210 (Hitachi, Tokyo, Japan) in 2013–2018, and Labospect 008AS (Hitachi, Tokyo, Japan) in 2019–2020. The low-density lipoprotein cholesterol (LDL-C) levels were calculated by Friedewald’s equation if the TG levels were <400 mg/dL [21].

### 2.4. Definition of Obesity and CVDRFs

The participants were classified into 3 groups based on BMI percentile for adjusting age and sex: normal weight, overweight, and obesity. Overweight and obesity were defined as a BMI of ≥85th to <95th percentile and of ≥95th percentile, respectively. Parental obesity was defined as a BMI of ≥30 kg/m^2^. High SBP and DBP were defined as SBP and DBP of ≥95th percentile, adjusted for age, sex, and height, for participants aged < 16 years, and SBP of ≥140 mmHg and DBP of ≥90 mmHg for participants aged 16–18 years, respectively, using blood pressure reference data for Korean children and adolescents [20]. Parental hypertension was diagnosed when SBP was ≥140 mmHg and/or DBP was ≥90 mmHg, or they were taking antihypertensive medication. The serum levels of total cholesterol ≥ 200 mg/dL, TG ≥ 130 mg/dL, HDL-C < 40 mg/dL, LDL-C ≥ 130 mg/dL, and fasting glucose ≥ 100 mg/dL were assigned as abnormal according to the pediatric guideline for cardiovascular health [22]. Parental diabetes mellitus was diagnosed when hemoglobin A1c was ≥6.5%, or fasting glucose was ≥126 mg/dL, or the subject was taking diabetic medication or insulin therapy.

### 2.5. Statistical Analyses

Sample weights were used for the analyses because of a stratified and multi-stage clustered sampling design of KNHANES. The sample weights are determined using a combination of probability of selection, nonresponse adjustment, and post-stratification adjustment methods to guarantee that survey data from KNHANES appropriately reflect the target population. Categorical variables were expressed as weighted percentages with standard errors and continuous variables were presented as weighted means with standard errors. We assessed normality of the variables using the Kolmogorov–Smirnov and Shapiro–Wilk tests. We used the analysis of covariance with the least significant difference test to compare age-adjusted mean values of BMI, lipid profiles, fasting glucose, and age- and height-adjusted mean values of blood pressure between boys and girls and between participants with normal weight, overweight, and obesity. We estimated the multivariable-adjusted prevalence of CVDRFs by logistic regression analysis using R statistical programming language. The data were analyzed separately for each sex because sex is known to influence the relationship between obesity and CVDRFs [17,23,24]. The associations of BMI z-score with age-adjusted lipid levels, fasting glucose levels, and age- and height-adjusted blood pressure values were analyzed via multiple linear regression analysis. Logistic regression analysis was used to estimate the age-adjusted odds ratios (ORs) with 95% confidence intervals (CIs) for associations between obesity and CVDRFs for each sex. In addition, to assume the age at which these relationships were shown, the same analyses were conducted in three age groups for each sex: early (10–12 years); middle (13–15 years); and late (16–18 years) adolescence. Statistical analyses were assessed using IBM SPSS Statistics version 20.0 (IBM Corp, Armonk, NY, USA) with integration plug-in for R. The version of R used in this study was 2.12.1. Statistical significance was set at *p* value < 0.05.

## 3. Results

### 3.1. Study Population

The characteristic of 8149 adolescents (4314 boys and 3835 girls) are shown in Table 1. All the variables were normally distributed (*p* values for all the variables in Kolmogorov–Smirnov and Shapiro–Wilk tests were > 0.05). The mean values for BMI were higher in boys than girls. The mean values of SBP, DBP, and fasting glucose were higher in boys than girls, whereas the mean levels of total cholesterol, TG, HDL–C, and LDL–C were higher in girls than in boys. The prevalence of high SBP, high DBP, low HDL–C, and high fasting glucose levels was higher in boys than in girls, whereas the prevalence of high total cholesterol and high LDL–C levels was higher in girls than in boys.

### 3.2. Association of BMI z-Score with CVDRFs

BMI z-score had a significant linear correlation with SBP, DBP, total cholesterol, TG, HDL-C, LDL-C, and fasting glucose levels in both boys and girls (Table 2).

### 3.3. Factors Affecting CVDRFs: Dietary Intake, Physcal Activity, and Parental CVDRFs

There were no significant differences in dietary intake or physical activity between the normal weight, overweight, and obesity groups in boys (Table 3). Significant differences in dietary intake were not observed between the normal weight, overweight, or obesity groups in girls either. Among physical activity, moderate- and vigorous-exercise times, but not walking time, were shorter in the overweight and obesity groups than in the normal weight group in girls.

The prevalences of all parental CVDRFs (paternal obesity, paternal hypertension, paternal diabetes mellitus, maternal obesity, maternal hypertension, and maternal diabetes mellitus) were higher in the obesity group than in the normal weight group, both in boys and girls. The prevalences of father with hypertension in boys, and father with hypertension, father with obesity, and mother with obesity in girls were higher in the overweight group than in the normal weight group.

### 3.4. Comparison of CVDRFs by Weight Status

The mean values of systolic and diastolic blood pressure were higher in boys and girls with overweight and obesity than those with normal weight (Table 4). Both boys and girls with overweight and obesity had higher mean total cholesterol, TG, LDL-C, and fasting glucose levels, as well as lower mean HDL-C levels, than those with normal weight.

There was a higher prevalence of high blood pressure and adverse levels of total cholesterol, TG, HDL-C, and LDL-C in boys with overweight and obesity than boys with normal weight (Table 5). Boys with normal weight and those with overweight did not vary in the prevalence of high fasting glucose levels. There was a higher prevalence of high blood pressure as well as adverse levels of total cholesterol, TG, HDL-C, LDL-C, and fasting glucose in girls with obesity than in girls with normal weight. Girls with normal weight and those with overweight did not vary in the prevalence of high SBP and DBP, high LDL-C and high total cholesterol and fasting glucose levels.

### 3.5. ORs for CVDRFs by Weight Status

**Figure 1:**
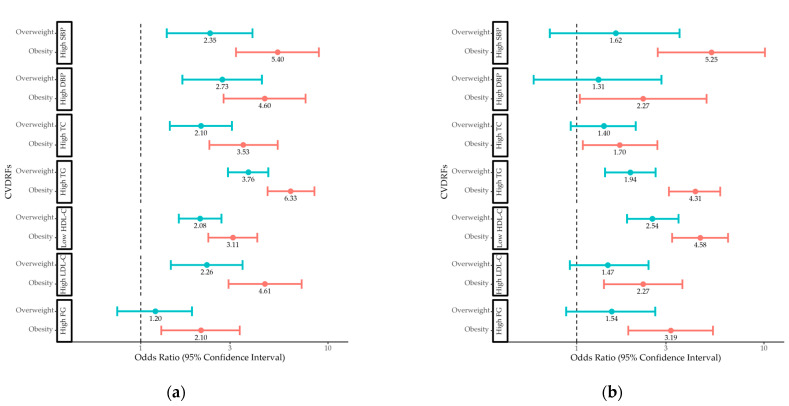
Odds ratios for CVDRFs in (**a**) boys and (**b**) girls with overweight and obesity compared to those with normal weight. CVDRF, cardiovascular disease risk factor; SBP, systolic blood pressure; DBP, diastolic blood pressure; TC, total cholesterol; TG, triglyceride; HDL-C, high-density lipoprotein cholesterol; LDL-C, low-density lipoprotein cholesterol; FG, fasting glucose.

The age-adjusted ORs for CVDRF by sex and weight status are shown in Figure 1. Boys and girls with obesity had increased odds of all CVDRFs. Boys with overweight had increased odds of high blood pressure and adverse lipid levels, whereas girls with overweight had increased odds of adverse TG and HDL-C levels.

### 3.6. ORs for CVDRFs by Weight Status in Different Age Groups

**Figure 2:**
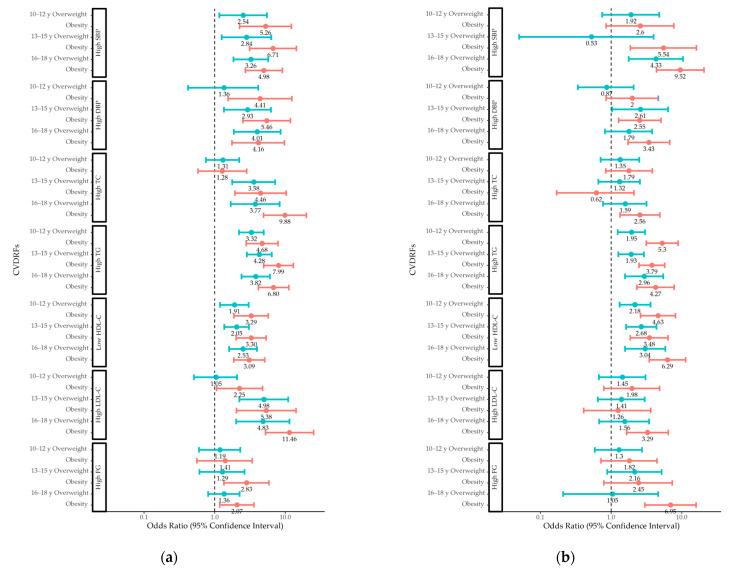
Odds ratios for CVDRFs in (**a**) boys and (**b**) girls with overweight and obesity compared to those with normal weight in different age groups. CVDRF, cardiovascular disease risk factor; SBP, systolic blood pressure; DBP, diastolic blood pressure; TC, total cholesterol; TG, triglyceride; HDL-C, high-density lipoprotein cholesterol; LDL-C, low-density lipoprotein cholesterol; FG, fasting glucose.

Boys who were overweight or obese had increased odds of high SBP and adverse TG and HDL-C levels in early, middle, and late adolescence, as well as increased odds of total cholesterol levels in middle and late adolescence (Figure 2). High DBP and high LDL-C levels in boys were associated with obesity in early, middle, and late adolescence, while they were associated with overweight in middle and late adolescence. High fasting glucose levels in boys were linked to obesity in middle and late adolescence, but they were not linked to adolescent overweight.

Girls who were overweight or obese had increased odds of adverse TG and HDL-C levels in early, middle, and late adolescence. Obesity in girls was associated with high SBP and DBP in middle and late adolescence, while being overweight in girls was associated with high SBP in late adolescence and high DBP in middle adolescence. High total cholesterol, high LDL-C, and high fasting glucose levels in girls were linked to obesity in late adolescence, but they were not linked to being overweight.

## 4. Discussion

In the present study, we investigated the association between adolescent obesity and CVDRFs. Compared to normal weight adolescents, adolescents with obesity had an association with every CVDRF, and overweight adolescents exhibited an association with some of them as well. The timing of the association between weight status and each CVDRF varied according to weight status variable, CVDRF variable, and sex. Among CVDRFs, high TG and low HDL-C levels were linked to being overweight and obese in both boys and girls from early to late adolescence. Other CVDRFs, such as high SBP and DBP, high total cholesterol and LDL-C levels, and high fasting glucose levels, are likely to present earlier in boys than girls and in adolescents with obesity than in adolescents with overweight.

Previous research has found a link between childhood obesity and high blood pressure, which was confirmed in this study [13,16,23,25]. In a study conducted by Lambert et al., both boys and girls with obesity had at least three times the odds of borderline or unfavorable SBP compared to those with normal weight (OR 3.1 [95% CI 2.1–5.2] and 4.7 [95% CI 2.9–7.7], respectively) [16]. In a 5-year cohort study of 1267 adolescents conducted by Dasgupta et al., high SBP was associated with a BMI of ≥ 85th in both boys and girls (OR 3.04 [1.80–5.13] and 2.25 [1.27–4.00], respectively) [25]. The present study also found that the association between being overweight/obese and hypertension develops earlier in boys than in girls. These findings might be explained by sex differences in lifestyle behaviors. High systolic blood pressure in teenagers was influenced by both increases in sedentary behavior and decreases in physical activity, even when overweight and obesity themselves were considered [25]. In a cross-sectional study of adolescents with severe obesity, boys were more likely to spend more time watching screens and had similar levels of physical activity to girls [26]. Differences in the distribution of excess fat across the sexes may also affect how soon a relationship between weight status and hypertension occurs. Visceral fat played an important role in adiposity-related hypertension in both adults and children [27,28,29]. During and after puberty, boys typically accumulated more visceral fat, whereas girls typically accumulate more total body fat and subcutaneous fat [30].

The present study demonstrated an association between obesity and dyslipidemia in male and female adolescents. It was consistent with previous studies, but there were some disparities in lipid variables based on the sex and weight status variables [11,13,16,17,31,32]. Freedman et al. demonstrated that children aged 5 to 17 years with a BMI of ≥95th percentile had greater odds of all adverse lipid levels compared to children with normal weight (OR 2.4 [95% CI 2.0–3.0], 7.1 [95% CI 5.8–8.6], 3.4 [95% CI 2.8–4.2], and 3.0 [95% CI 2.4–3.6] of adverse total cholesterol, TG, HDL-C, and LDL-C levels, respectively) [11]. Skinner et al. demonstrated that obesity was associated with all adverse total cholesterol, TG, and HDL-C levels (OR 2.4, 3.8, and 6.0, respectively), and overweight was associated with low HDL-C levels (OR 3.3) in children aged 6 to 17 years old [14]. In a Canadian population-based study conducted by Lambert et al, obesity was associated with borderline or unfavorable total cholesterol, TG, HDL-C, and LDL-C levels in boys (OR 2.3 [1.5–3.7], 16.1 [6.5–40.0], 3.0 [1.8–5.1], and 2.5 [1.6–3.9], respectively) and borderline or unfavorable TG and HDL-C levels in girls (OR 3.9 [1.5–10.2] and 3.6 [1.9–6.9], respectively) [16]. The mechanism underlying sex differences in the obesity/dyslipidemia relationship was not fully understood. These differences could be attributed to sex differences in the accumulation of visceral adipose tissue during puberty [30]. According to an adult study conducted by Lemieux et al., adjustment of abdominal visceral fat area removed the differences for TG and apolipoprotein level between men and women [33]. Dietary intake and sex hormone disparity also could be related to sex difference in the obesity/dyslipidemia relationship. In a study of the Australian National Nutrition Survey, McNaughton et al. reported male adolescents were more likely to have high fat and sugar dietary patterns than female adolescents [34]. In a longitudinal observational study, Morrison et al. showed a rise in free testosterone was associated adverse lipid levels in male adolescents [35].

High fasting glucose levels were linked to obesity but not to being overweight in adolescents: these links emerged earlier in boys than in girls. These findings were accordance with previous studies showing a greater prevalence of prediabetes and type 2 diabetes in children and adolescents with obesity [16,36,37,38]. In a study based on NHANES, Li et al. demonstrated adolescents with severe obesity had a higher likelihood of having high FG than normal weight adolescents, but neither moderately obese nor overweight adolescents did (OR 2.68 [95% CI 1.81–3.99], 1.49 [95% CI 0.93–2.37] and 1.19 [95% CI 0.83–1.71] in adolescents with severe obesity, moderate obesity, and overweight) [13]. In a study of 2144 Danish children and adolescents, Kloppenborg et al. demonstrated participants of a BMI ≥ 90 percentile have higher prevalence of impaired fasting glucose (≥100 mg/dL) than normal weight participants (14.3% vs. 4.5%) [39]. Impaired fasting glucose is one of diagnostic criteria for prediabetes and closely related to changes in β cell glucose sensitivity and liver insulin sensitivity [40,41]. Adipose tissue releasing nonesterified fatty acids and other mediators cause pancreatic β cell dysfunction and insulin resistance, which leads to prediabetes and diabetes mellitus [42,43].

The findings of the present study demonstrated that adolescent obesity was associated with all CVDRFs, with some CVDRF variables showing this association as early as 10–12 years old. These results emphasize the significance of early detection of obesity, and physicians can use routine weight status assessments as a method to identify teenagers who are at a higher risk of developing CVDRFs. In addition, the present study revealed that the associations between obesity and CVDRFs were shown to appear at different ages, depending on the degree of obesity, sex, and CVDRF variable. When considering limited medical resources and costly and labor-intensive obesity treatments for adolescent obesity, identifying the age at which obesity begins to show an association with certain CVDRFs can help efficiently screen and manage CVDRFs as well as prevent future cardiovascular disease.

This study had several limitations. First, because this was not a longitudinal cohort study, we were unable to demonstrate a causal relationship between obesity and CVDRFs and follow the relationship between obesity and CVDRFs in adulthood. Thus, this study only suggests the specific period of age when the relationship between obesity and CVDRFs was observed. Second, because this study only included youths from 10 to 18 years of age, we could not identify the trend of the relationship between being overweight/obese and CVDRFs before 10 years of age. A longitudinal cohort study including participants under the age of 10 years is required to identify the exact timing of when the relationship between obesity and CVDRFs begins.

## 5. Conclusions

Overweight and obesity are associated with CVDRFs in Korean adolescents. The associations between obesity and CVDRFs have been observed at different periods of youth, depending on the degree of obesity, CVDRF variables, and sex. Identification of when these associations are observed may allow for screening and management of CVDRFs as well as prevention of future cardiovascular disease.

## Figures and Tables

**Table 1:** Study population.

Variable	Boys	Girls	*p* Value
Age (years)	13.7 (2.5) (n = 4314)	13.7 (2.5) (n = 3835)	0.942
BMI * (kg/m^2^)	20.77 (0.05) (n = 4314)	19.99 (0.06) (n = 3835)	<0.001
SBP † (mmHg)	108.5 (10.7) (n = 4301)	104.1 (9.3) (n = 3819)	<0.001
DBP † (mmHg)	66.8 (9.7) (n = 4301)	65.5 (8.5) (n = 3819)	<0.001
Total cholesterol * (mg/dL)	154.2 (26.8) (n = 3819)	162.2 (25.8) (n = 3355)	<0.001
Triglyceride * (mg/dL)	84.9 (53.1) (n = 3820)	87.7 (49.5) (n = 3355)	0.023
HDL-C * (mg/dL)	50.6 (11.0) (n = 3820)	53.3 (11.0) (n = 3355)	<0.001
LDL-C * (mg/dL)	86.6 (23.1) (n = 3819)	91.5 (22.4) (n = 3355)	<0.001
Fasting glucose * (mg/dL)	90.0 (7.3) (n = 3789)	88.9 (9.1) (n = 3338)	<0.001
Energy intake * (kcal/d)	2305.4 (11.1) (n = 3931)	1838.2 (11.7) (n = 3532)	<0.001
Protein intake * (g/d)	84.3 (0.5) (n = 3931)	65.1 (0.6) (n = 3532)	<0.001
Fat intake * (g/d)	62.0 (0.5) (n = 3931)	48.6 (0.5) (n = 3532)	<0.001
Carbohydrate intake * (g/d)	347.8 (1.6) (n = 3931)	283.4 (1.7) (n = 3532)	<0.001
Sodium intake * (mg/d)	4082.3 (28.1) (n = 3931)	3169.5 (29.6) (n = 3532)	<0.001
Walking * (hr/wk)	5.4 (0.1) (n = 3150)	4.9 (0.1) (n = 2778)	0.003
Moderate exercise * (hr/wk)	3.0 (0.1) (n = 2057)	2.2 (0.1) (n = 1518)	<0.001
Vigorous exercise * (hr/wk)	3.5 (0.1) (n = 2380)	1.8 (0.1) (n = 1562)	<0.001
Prevalence of CVDRFs *			
High SBP (%)	2.3 (0.2)	1.5 (0.2)	0.012
High DBP (%)	2.5 (0.2)	1.6 (0.2)	0.005
High total cholesterol (%)	5.4 (0.4)	8.1 (0.4)	<0.001
High triglyceride (%)	13.3 (0.5)	13.1 (0.6)	0.849
Low HDL-C (%)	15.4 (0.5)	10.0 (0.6)	<0.001
High LDL-C (%)	3.9 (0.3)	5.6 (0.4)	<0.001
High fasting glucose (%)	5.1 (0.3)	3.9 (0.4)	0.010

Data are expressed as mean (standard error) or percentage (standard error). Analysis of covariance was used to compare variables between boys and girls, while logistic regression analysis with the R statistical programming language was used to compare cardiovascular disease risk factors between boys and girls. * Adjusted for age. † Adjusted for age and height. BMI, body mass index; SBP, systolic blood pressure; DBP, diastolic blood pressure; HDL-C, high-density lipoprotein cholesterol; LDL-C, low-density lipoprotein cholesterol; CVDRF, cardiovascular disease risk factor; n, number of participants evaluated.

**Table 2:** Association between BMI z-score and CVDRFs.

Sex	Variable	*β*	SE	s.b	*p* Value
Boys	SBP * (mmHg)	3.289	0.147	0.301	<0.001
	DBP * (mmHg)	1.089	0.140	0.110	<0.001
	Total cholesterol † (mg/dL)	6.086	0.428	0.220	<0.001
	Triglyceride † (mg/dL)	16.319	0.845	0.298	<0.001
	HDL-C † (mg/dL)	–2.762	0.174	–0.244	<0.001
	LDL-C † (mg/dL)	5.588	0.371	0.234	<0.001
	Fasting glucose † (mg/dL)	1.045	0.119	0.139	<0.001
Girls	SBP * (mmHg)	2.180	0.149	0.229	<0.001
	DBP * (mmHg)	0.755	0.135	0.088	<0.001
	Total cholesterol † (mg/dL)	2.470	0.452	0.094	<0.001
	Triglyceride † (mg/dL)	10.816	0.846	0.214	<0.001
	HDL-C † (mg/dL)	–2.776	0.188	-0.246	<0.001
	LDL-C † (mg/dL)	3.162	0.392	0.138	<0.001
	Fasting glucose † (mg/dL)	0.980	0.159	0.105	<0.001

Multiple linear regression analysis was used. * Adjusted for age and height. † Adjusted for age. BMI, body mass index; CVDRF, cardiovascular disease risk factor; SBP, systolic blood pressure; DBP, diastolic blood pressure; HDL-C, high-density lipoprotein cholesterol; LDL-C, low-density lipoprotein cholesterol; ***β***, unstandardized coefficients; SE, standard error; s.b, standardized coefficients

**Table 3:** Comparison of dietary intake, physical activity, and parental CVDRFs by weight status.

Sex	Variable	Normal weight	Overweight	Obesity	*p* Value †	*p* Value ‡	*p* Value §
Boys	Energy intake * (kcal/d)	2258.1 (37.0)	2309.4 (3.5)	2318.8 (39.4)	0.193	0.262	0.822
	Protein intake * (g/d)	83.8 (0.6)	85.8 (1.8)	86.6 (1.9)	0.296	0.160	0.748
	Fat intake * (g/d)	61.8 (1.6)	61.9 (1.7)	62.0 (0.6)	0.949	0.916	0.987
	Carbohydrate intake * (g/d)	324.4 (5.3)	347.5 (5.7)	349.5 (2.0)	0.091	0.108	0.740
	Sodium intake * (mg/d)	4052.8 (33.8)	4171.7 (92.5)	4210.5 (98.5)	0.227	0.130	0.774
	Walking * (hr/wk)	5.6 (0.1)	5.0 (0.2)	4.7 (0.4)	0.136	0.060	0.703
	Moderate exercise * (hr/wk)	3.1 (0.1)	3.0 (0.4)	2.9 (0.3)	0.892	0.631	0.806
	Vigorous exercise * (hr/wk)	3.9 (0.3)	3.5 (0.3)	3.4 (0.1)	0.448	0.192	0.711
	Paternal obesity * (%)	1.3 (0.2)	1.8 (0.5)	5.2 (0.5)	0.412	<0.001	<0.001
	Paternal hypertension * (%)	28.8 (1.1)	37.1 (3.0)	38.4 (2.8)	0.010	0.001	0.753
	Paternal diabetes mellitus * (%)	9.3 (1.0)	13.3 (2.8)	16.2 (2.4)	0.168	0.007	0.433
	Maternal obesity * (%)	1.6 (0.2)	2.5 (0.6)	3.9 (0.5)	0.134	<0.001	0.061
	Maternal hypertension * (%)	9.7 (0.6)	13.2 (1.7)	16.0 (1.7)	0.055	<0.001	0.246
	Maternal diabetes mellitus * (%)	3.2 (1.6)	3.7 (0.6)	8.7 (1.4)	0.754	0.001	0.009
Girls	Energy intake * (kcal/d)	1793.9 (35.5)	1822.1 (35.8)	1844.9 (11.1)	0.575	0.171	0.543
	Protein intake * (g/d)	64.7 (1.7)	65.0 (0.5)	66.4 (1.7)	0.838	0.466	0.435
	Fat intake * (g/d)	47.4 (1.5)	48.5 (1.5)	48.7 (0.5)	0.590	0.398	0.908
	Carbohydrate intake * (g/d)	273.8 (5.4)	281.4 (5.4)	284.6 (1.7)	0.322	0.056	0.569
	Sodium intake * (mg/d)	3119.4 (93.7)	3173.7 (93.0)	3176.5 (29.1)	0.681	0.561	0.977
	Walking * (hr/wk)	5.4 (0.4)	5.0 (0.4)	4.8 (0.1)	0.472	0.224	0.797
	Moderate exercise * (hr/wk)	3.2 (0.3)	2.1 (0.1)	1.8 (0.3)	0.001	0.001	0.313
	Vigorous exercise * (hr/wk)	2.5 (0.3)	1.8 (0.3)	1.7 (0.1)	0.049	0.017	0.752
	Paternal obesity * (%)	1.1 (0.2)	4.7 (0.6)	5.7 (0.6)	<0.001	<0.001	0.235
	Paternal hypertension * (%)	28.1 (1.1)	34.6 (3.3)	36.6 (3.2)	0.044	0.011	0.661
	Paternal diabetes mellitus * (%)	8.2 (1.0)	11.4 (2.8)	16.0 (3.0)	0.283	0.014	0.256
	Maternal obesity * (%)	1.5 (0.2)	4.7 (0.7)	8.3 (0.7)	<0.001	<0.001	<0.001
	Maternal hypertension * (%)	9.2 (0.6)	11.6 (1.9)	16.0 (1.9)	0.228	0.001	0.106
	Maternal diabetes mellitus * (%)	3.8 (0.6)	6.4 (1.9)	11.8 (1.8)	0.196	<0.001	0.043

Analysis of covariance was used to compare variables between normal weight, overweight, and obesity. Data are expressed as mean (standard error) or percentage (standard error). * Adjusted for age. † p value for normal weight vs. overweight. ‡ p value for normal weight vs. obesity. § p value for overweight vs. obesity.

**Table 4:** Comparison of blood pressure, lipid profiles, and fasting glucose by weight status for each sex.

Sex	Variable	Normal Weight	Overweight	Obesity	*p* Value ‡	*p* Value §	*p* Value ¶
Boys	SBP * (mmHg)	107.2 (0.2)	112.6 (0.4)	116.3 (0.6)	<0.001	<0.001	<0.001
	DBP * (mmHg)	66.2 (0.2)	68.7 (0.4)	69.9 (0.5)	<0.001	<0.001	0.049
	Total cholesterol † (mg/dL)	151.8 (0.5)	161.9 (1.2)	169.1 (1.6)	<0.001	<0.001	<0.001
	Triglyceride † (mg/dL)	77.8 (0.9)	110.4 (2.3)	123.8 (3.2)	<0.001	<0.001	<0.001
	HDL-C † (mg/dL)	51.8 (0.2)	46.5 (0.5)	44.6 (0.7)	<0.001	<0.001	0.020
	LDL-C † (mg/dL)	84.5 (0.4)	93.3 (1.0)	99.8 (1.4)	<0.001	<0.001	<0.001
	Fasting glucose † (mg/dL)	89.6 (0.1)	91.2 (0.3)	92.8 (0.5)	<0.001	<0.001	<0.001
Girls	SBP * (mmHg)	103.2 (0.2)	107.0 (0.4)	109.6 (0.6)	<0.001	<0.001	<0.001
	DBP * (mmHg)	65.2 (0.1)	66.6 (0.4)	67.9 (0.5)	<0.001	<0.001	0.048
	Total cholesterol † (mg/dL)	160.5 (0.5)	162.9 (1.3)	170.7 (1.7)	0.031	<0.001	<0.001
	Triglyceride † (mg/dL)	83.4 (0.9)	98.9 (2.5)	122.9 (3.3)	<0.001	<0.001	<0.001
	HDL-C † (mg/dL)	54.3 (0.2)	49.7 (0.6)	45.8 (0.7)	<0.001	<0.001	<0.001
	LDL-C † (mg/dL)	90.5 (0.4)	93.8 (1.2)	100.4 (1.5)	0.007	<0.001	<0.001
	Fasting glucose † (mg/dL)	88.4 (0.2)	90.3 (0.5)	92.0 (0.6)	<0.001	<0.001	<0.001

Analysis of covariance was used to compare variables between normal weight, overweight, and obesity. Data are expressed as mean (standard error). * Adjusted for age, height, dietary intake, physical activity, and parental CVDRFs. † Adjusted for age, dietary intake, physical activity, and parental CVDRFs. ‡ *p* value for normal weight vs. overweight. § *p* value for normal weight vs. obesity. ¶ *p* value for overweight vs. obesity. SBP, systolic blood pressure; DBP, diastolic blood pressure; HDL-C, high-density lipoprotein cholesterol; LDL-C, low-density lipoprotein cholesterol.

**Table 5:** Prevalence of CVDRFs by age, sex, and weight status adjusted for dietary intake, physical activity, and parental CVDRFs.

Sex	Risk Factor Variable	Weight Status	All Ages			10–12 y			13–15 y			16–18 y		
			Total (n)	Prevalence % (SE)	*p* Value	Total (n)	Prevalence % (SE)	*p* Value	Total (n)	Prevalence % (SE)	*p* Value	Total (n)	Prevalence % (SE)	*p* Value
Boys	High SBP	Normal weight	3457	1.7 (0.3)		1313	1.8 (0.4)		1217	1.9 (0.5)		927	1.6 (0.3)	
		Overweight	557	3.8 (0.7)	0.003 *	247	4.6 (1.0)	0.007 *	180	5.0 (1.2)	0.018 *	130	4.6 (0.8)	0.001 *
		Obesity	288	8.0 (0.9)	<0.001 ^†^	97	8.9 (1.6)	<0.001 ^†^	100	10.5 (1.7)	<0.001 ^†^	91	6.9 (1.0)	<0.001 ^†^
					<0.001 ^‡^			0.035 ^‡^			0.011 ^‡^			0.127 ^‡^
	High DBP	Normal weight	3457	1.9 (0.3)		1313	1.3 (0.3)		1217	2.2 (0.5)		927	4.6 (1.2)	
		Overweight	557	5.6 (0.7)	<0.001 *	247	1.8 (0.8)	0.552 *	180	6.1 (1.3)	0.004 *	130	17.8 (3.2)	<0.001 *
		Obesity	288	8.0 (0.9)	<0.001 ^†^	97	5.4 (1.3)	0.002 ^†^	100	9.8 (1.8)	<0.001 ^†^	91	17.6 (3.8)	0.001 ^†^
					0.049 ^‡^			0.029 ^‡^			0.193 ^‡^			0.989 ^‡^
	High TC	Normal weight	3081	4.3 (0.4)		1114	7.9 (0.8)		1129	2.4 (0.5)		838	2.5 (0.7)	
		Overweight	487	8.8 (1.0)	<0.001 *	201	9.6 (1.9)	0.422 *	167	8.2 (1.4)	<0.001 *	119	8.7 (1.8)	0.002 *
		Obesity	252	13.1 (1.4)	<0.001 ^†^	83	8.7 (3.0)	0.787 ^†^	84	11.5 (2.0)	<0.001 ^†^	85	19.2 (2.2)	<0.001 ^†^
					0.021 ^‡^			0.836 ^‡^			0.258 ^‡^			<0.001 ^‡^
	High TG	Normal weight	3081	9.2 (0.6)		1114	8.9 (1.0)		1129	8.6 (1.0)		838	10.0 (1.2)	
		Overweight	487	27.5 (1.5)	<0.001 *	201	23.8 (2.3)	<0.001 *	167	29.3 (2.5)	<0.001 *	119	29.4 (3.1)	<0.001 *
		Obesity	252	39.6 (2.1)	<0.001 ^†^	83	30.4 (3.6)	<0.001 ^†^	84	45.2 (3.6)	<0.001 ^†^	85	43.3 (3.7)	<0.001 ^†^
					<0.001 ^‡^			0.218 ^‡^			<0.001^‡^			0.008 ^‡^
	Low HDL-C	Normal weight	3081	13.0 (0.6)		1114	8.9 (0.9)		1129	15.4 (1.1)		838	14.7 (1.3)	
		Overweight	487	23.9 (1.6)	<0.001 *	201	15.0 (2.2)	0.008 *	167	26.6 (3.0)	<0.001 *	119	30.2 (3.5)	<0.001 *
		Obesity	252	31.4 (2.3)	<0.001 ^†^	83	22.8 (3.4)	<0.001 ^†^	84	37.1 (4.3)	<0.001 ^†^	85	34.3 (4.1)	<0.001 ^†^
					0.018 ^‡^			0.139 ^‡^			0.068 ^‡^			0.536 ^‡^
	High LDL-C	Normal weight	3081	2.9 (0.3)		1114	5.3 (0.7)		1129	1.5 (0.5)		838	1.8 (0.6)	
		Overweight	487	6.6 (0.9)	<0.001 *	201	5.2 (1.6)	0.921 *	167	6.8 (1.2)	<0.001 *	119	7.8 (1.7)	0.001 *
		Obesity	252	11.3 (1.2)	<0.001 ^†^	83	9.7 (2.5)	0.095 ^†^	84	7.8 (1.7)	<0.001^†^	85	16.4 (2.0)	<0.001 ^†^
					0.024 ^‡^			0.385 ^‡^			0.637 ^‡^			<0.001 ^‡^
	High FG	Normal weight	3055	4.7 (0.4)		1100	5.4 (0.7)		1123	4.7 (0.7)		832	4.6 (0.5)	
		Overweight	483	5.4 (1.0)	0.551 *	198	6.4 (1.6)	0.577 *	166	5.6 (1.7)	0.644 *	119	5.6 (1.2)	0.418 *
		Obesity	251	9.7 (1.4)	0.001 ^†^	82	7.2 (2.6)	0.509 ^†^	84	13.0 (2.5)	0.001 ^†^	85	9.3 (1.6)	0.004 ^†^
					0.041 ^‡^			0.719 ^‡^			0.024 ^‡^			0.048 ^‡^
Girls	High SBP	Normal weight	3160	1.1 (0.2)		1222	2.1 (0.4)		1068	1.3 (0.4)		870	2.0 (0.6)	
		Overweight	417	1.9 (0.6)	0.253 *	161	4.1 (1.2)	0.114 *	149	0.6 (1.0)	0.563 *	107	7.7 (1.7)	0.002 *
		Obesity	244	5.3 (0.8)	<0.001 ^†^	80	4.7 (1.7)	0.148 ^†^	78	6.8 (1.4)	<0.001 ^†^	86	14.7 (1.9)	<0.001 ^†^
					0.003 ^‡^			0.722 ^‡^			<0.001 ^‡^			0.011 ^‡^
	High DBP	Normal weight	3160	1.5 (0.2)		1222	4.8 (0.7)		1068	3.2 (0.5)		870	5.0 (0.8)	
		Overweight	417	2.0 (0.6)	0.490 *	161	4.0 (1.8)	0.678 *	149	7.5 (2.1)	0.049 *	107	8.5 (2.3)	0.153 *
		Obesity	244	3.5 (0.8)	0.021 ^†^	80	9.2 (2.5)	0.081 ^†^	78	7.8 (1.5)	0.004 ^†^	86	14.7 (2.6)	<0.001 ^†^
					0.207 ^‡^			0.120 ^‡^			0.738 ^‡^			0.165 ^‡^
	High TC	Normal weight	2770	7.6 (0.5)		999	8.2 (0.9)		975	7.0 (0.8)		796	7.7 (1.0)	
		Overweight	369	10.2 (1.5)	0.100 *	136	11.0 (2.5)	0.284 *	136	8.1 (2.2)	0.659 *	97	11.5 (2.9)	0.216 *
		Obesity	216	12.1 (1.9)	0.024 ^†^	67	13.9 (3.5)	0.107 ^†^	68	4.8 (3.2)	0.489 ^†^	81	17.5 (3.2)	0.003 ^†^
					0.446 ^‡^			0.583 ^‡^			0.461 ^‡^			0.298 ^‡^
	High TG	Normal weight	2770	10.6 (0.6)		999	14.8 (1.2)		975	8.4 (0.7)		796	6.4 (1.0)	
		Overweight	369	19.1 (1.8)	<0.001 *	136	26.1 (3.3)	0.001 *	136	15.7 (2.0)	0.001 *	97	16.0 (2.8)	0.001 *
		Obesity	216	32.8 (2.3)	<0.001 ^†^	67	46.4 (4.6)	<0.001 ^†^	68	26.0 (2.5)	<0.001 ^†^	81	23.4 (3.1)	<0.001 ^†^
					<0.001 ^‡^			<0.001 ^‡^			0.003 ^‡^			0.049 ^‡^
	Low HDL-C	Normal weight	2770	7.6 (0.6)		999	9.1 (1.0)		975	8.3 (1.0)		796	5.4 (0.9)	
		Overweight	369	16.6 (1.6)	<0.001 *	136	16.7 (2.7)	0.008 *	136	19.2 (2.6)	<0.001 *	97	13.8 (2.7)	0.003 *
		Obesity	216	27.3 (2.0)	<0.001 ^†^	67	31.1 (3.8)	<0.001 ^†^	68	24.5 (3.8)	<0.001 ^†^	81	26.4 (2.9)	<0.001 ^†^
					<0.001 ^‡^			0.004 ^‡^			0.134 ^‡^			<0.001 ^‡^
	High LDL-C	Normal weight	2770	5.0 (0.6)		999	4.8 (0.7)		975	4.7 (0.7)		796	5.5 (0.9)	
		Overweight	369	7.0 (1.6)	0.333 *	136	7.3 (2.0)	0.243 *	136	6.0 (1.9)	0.512 *	97	8.6 (2.5)	0.244 *
		Obesity	216	11.1 (2.0)	<0.001 ^†^	67	9.2 (2.8)	0.125 ^†^	68	6.7 (2.7)	0.465 ^†^	81	15.8 (2.8)	<0.001 ^†^
					0.213 ^‡^			0.543 ^‡^			0.715 ^‡^			0.065 ^‡^
	High FG	Normal weight	2756	3.1 (0.4)		990	5.1 (0.7)		970	2.3 (0.5)		796	2.0 (0.6)	
		Overweight	366	4.6 (1.0)	0.185 *	135	6.5 (2.0)	0.535 *	135	5.0 (1.5)	0.078 *	96	2.3 (1.7)	0.885 *
		Obesity	216	8.9 (1.3)	<0.001 ^†^	67	8.7 (2.8)	0.230 ^†^	69	5.9 (2.1)	0.092 ^†^	80	12.2 (1.9)	<0.001 ^†^
					0.013 ^‡^			0.445 ^‡^			0.625 ^‡^			<0.001 ^‡^

Logistic regression analysis with the R statistical programming language was used. Data are expressed as percentage (standard error). * *p* value for normal weight vs. overweight. ^†^ *p* value for normal weight vs. obesity. ^‡^ p value for overweight vs. obesity. CVDRF, cardiovascular disease risk factor; SBP, systolic blood pressure; DBP, diastolic blood pressure; TC, total cholesterol; TG, triglyceride; HDL-C, high-density lipoprotein cholesterol; LDL-C, low-density lipoprotein cholesterol; FG, fasting glucose; n, number of participants evaluate; SE, standard error.

## Data Availability

Data supporting reported results can be found via publicly available datasets (https://knhanes.kdca.go.kr/knhanes/sub03/sub03_02_05.do).
